# Contralateral Prophylactic Mastectomy (CPM) Consensus Statement from the American Society of Breast Surgeons: Data on CPM Outcomes and Risks

**DOI:** 10.1245/s10434-016-5443-5

**Published:** 2016-07-28

**Authors:** Judy C. Boughey, Deanna J. Attai, Steven L. Chen, Hiram S. Cody, Jill R. Dietz, Sheldon M. Feldman, Caprice C. Greenberg, Rena B. Kass, Jeffrey Landercasper, Valerie Lemaine, Fiona MacNeill, David H. Song, Alicia C. Staley, Lee G. Wilke, Shawna C. Willey, Katharine A. Yao, Julie A. Margenthaler

**Affiliations:** 1 Department of Surgery, Mayo Clinic, Rochester, MN USA; 2 Department of Surgery, David Geffen School of Medicine at UCLA, UCLA Health Burbank Breast Care, Burbank, CA USA; 3 Department of Surgery, OasisMD, San Diego, CA USA; 4 Department of Surgery, Memorial Sloan Kettering Cancer Center, New York, NY USA; 5 Department of Surgery, Case Western Reserve School of Medicine, Seidman Cancer Center, Cleveland, OH USA; 6 Department of Surgery, Columbia University, New York, NY USA; 7 Department of Surgery, University of Wisconsin, Madison, WI USA; 8 Department of Surgery, College of Medicine, The Pennsylvania State University, Hershey, PA USA; 9 Department of Surgery, Gundersen Health System, Lacrosse, WI USA; 10 Department of Surgery, Royal Marsden Hospital, London, UK; 11 Department of Surgery, University of Chicago, Chicago, IL USA; 12 Akari Healthcare, Charleston, MA USA; 13 Department of Surgery, Medstar Georgetown University Hospital, Washington, DC USA; 14 Department of Surgery, NorthShore University Health System, Evanston, IL USA; 15 Department of Surgery, Center for Advanced Medicine, Breast Health Center, St. Louis, MO USA

The American Society of Breast Surgeons (ASBrS) endorses the American Board of Internal Medicine’s Choosing Wisely campaign statement: “Don’t routinely perform a double mastectomy in patients who have a single breast with cancer.”^1^ However, women with a newly diagnosed unilateral breast cancer are increasingly opting for bilateral mastectomy. This has been seen in patients who are candidates for breast conservation who elect mastectomy as well as those requiring mastectomy for their index breast cancer.^2^ National rates of contralateral prophylactic mastectomy (CPM) in the United States have been increasing and this trend is continuing.^2^^–^4

There has been significant controversy surrounding this topic, and it has received attention from national societies as well as the media. Therefore, the ASBrS convened a panel of experts to develop a consensus statement on CPM. Initial literature review was performed and exchanged electronically by the panel, followed by an in-person meeting of the consensus group and polling of the membership of the ASBrS at the 2016 annual meeting. The consensus statement consists of two parts. This paper, part 1, outlines the data on the impact of CPM on cancer and noncancer outcomes, including risks of CPM and when CPM should be considered or discouraged. Part 2 outlines whether CPM utilization should be a quality measure, role of sentinel node biopsy for CPM, perspectives on CPM from patients and from providers in other countries, and counseling considerations for patients desiring CPM and includes a discussion template for providers to use with patients regarding CPM. The ASBrS Executive Committee reviewed and approved the statement. This consensus statement was developed to guide patient and physician discussion and should not affect insurance coverage.

The consensus group agreed that CPM should be discouraged for an average-risk woman with unilateral breast cancer. However, patient’s values, goals, and preferences should be included to optimize shared decision making when discussing CPM. The final decision whether or not to proceed with CPM is a result of the balance between benefits and risks of CPM and patient preference.

## Breast Conservation or Mastectomy

The consensus group recommends consideration of breast conservation for all patients who are appropriate candidates. Breast conservation is equivalent to mastectomy in survival outcome and has been the preferred treatment for early-stage breast cancer since the National Cancer Institute statement in 1991.^5^ Neoadjuvant chemotherapy and neoadjuvant endocrine therapy are highly successful in providing tumor size reduction to increase breast conservation rates, and oncoplastic approaches allow resection of larger tumors with reshaping and provide an excellent cosmetic outcome.^6^^–^9 Increase in use of breast conservation can decrease CPM rates. Complication rates have been shown to be lower with breast-conserving surgery and adjuvant radiation than with mastectomy and reconstruction.^10^

### Summary

The panel recommends advocating for breast conservation for all appropriately eligible patients and considering neoadjuvant systemic therapy and/or oncoplastic approaches to facilitate breast conservation where possible.

## Contralateral Prophylactic Mastectomy

For women who elect or require mastectomy for management of their index breast cancer, the option of removing the contralateral breast is often discussed. Multiple factors should be considered including family history, patient age, comorbidities, and tumor prognosis, as well as the initial plan for surgery, systemic therapy, and radiotherapy. The surgical consultation should include a detailed discussion of local treatment options, the risk of developing a contralateral breast cancer (CBC) and distant cancer recurrence, the options for managing a CBC, and a clear recommendation for or against CPM. CPM is never an emergency and is never mandatory; even for patients at the highest risk of CBC, in the absence of disease, close surveillance is always a reasonable alternative to surgery.

### CPM and Impact on Cancer Outcomes

#### Risk of CBC and Reduction of CBC with CPM

Among women with breast cancer, the absolute risk of developing a CBC exceeds that of the general population and is approximately 0.6 % per year in historic series.^11^ Because systemic adjuvant chemotherapy reduces this risk by about 20 %, tamoxifen by about 50 %, and aromatase inhibitors by about 60 %, the contemporary risk of developing a CBC is likely lower at 0.2–0.5 % per year for those undergoing adjuvant therapies.^12^ Survivorship bias must be taken into account when looking at factors associated with CBC because the only patients who develop a CBC are those who survive their first primary cancer.

Known factors contributing to the risk of developing CBC include individual patient factors (family history, gene mutation status, patient age, etc.) as well as treatment related to the index breast cancer (use of chemotherapy, hormone therapy, etc.). For known *BRCA1/2* mutation carriers, studies have shown a 30–40 % risk of CBC at 10 years, and the risk appears to continue beyond 10 years.^13^ The CBC risk for carriers of other genes such as *CHEK2, p53, PALB2, ATM,* and *NBN* is less well studied, and at this time there is insufficient evidence to support an increased CBC risk for these mutations. Early studies of *CHEK2* mutation carriers showed an increased risk for CBC, but future studies are warranted to verify this finding.^14^^,^^15^ Patients at higher risk for breast cancer, such as those who have undergone mantle radiation for Hodgkin lymphoma, may be at increased risk for CBC, but few studies have verified this.^16^ For patients with a significant family history with no proven gene mutation or in whom genetic testing was not done, the risk of CBC varies by number and degree of the relative(s) affected by breast cancer and the age of the patient. In the WECARE study, the 10-year cumulative risk of breast cancer for a 30-year-old woman with a first-degree relative is 14.7 % compared to a 50-year-old woman, whose risk is 6.7 % at 10 years.^17^ CBC risk varies by the hormone receptor status of the primary tumor. In the overview analysis, the risk of CBC was 0.4 % per year for estrogen receptor–positive patients and 0.5 % per year for estrogen receptor-negative patients.^18^ A Surveillance, Epidemiology, and End Results (SEER) study performed nearly 10 years ago showed that CBC risk has been decreasing 3 % per year since 1985 due in part to increased use of hormone therapy.^19^ Annual risks of CBC are highest in patients younger than 30 years and patients with multiple affected first- and second-degree relatives (annual risk 0.4–1.3 %).

There is strong evidence that CPM reduces the relative risk of cancer in the contralateral breast by 90 to 95 %; however, breast cancer risk is not completely eliminated with CPM. The absolute risk of developing cancer on that side after CPM ranges from 0 to 1.5 %.^20^^–^25

##### Summary

Risk of CBC for average-risk women with breast cancer is 0.1 to 0.6 % per year. CBC risk is higher for women diagnosed at a young age, those with a strong family history, and *BRCA* carriers.

#### Impact of CPM on Overall Survival and Disease-Free Survival

There are no prospective studies on the impact of CPM on overall survival (OS) and disease-free survival (DFS). In view of this, all published studies comparing OS and DFS for woman undergoing CPM with those not undergoing CPM have to be interpreted with caution, as they are retrospective and may contain known and unknown biases. Multiple retrospective studies have compared OS and DFS in women undergoing CPM compared to those not undergoing CPM. Some have shown a DFS benefit^21^^,^^24^^–^29 and some have shown an OS benefit to CPM.^21^^,^^27^^–^30 However, these studies cannot establish a causal link between CPM and survival. The Cochrane Database, a meta-analysis, and other studies indicate no difference in OS or breast cancer–specific survival and cite selection bias as the cause of superior outcomes in the studies that demonstrated improvement in survival.^22^^,^^31^^,^^32^ A recent SEER analysis examined CPM’s effect not only on all-cause and breast cancer mortality but also on noncancer mortality.^33^ This study showed a greater reduction in noncancer mortality for CPM than for all-cause or breast cancer mortality and illustrates how selection bias likely accounts for the survival benefit reported from CPM.

For those patients at the highest CBC risk, such as *BRCA* carriers, retrospective studies have shown that patients who underwent CPM had better OS than *BRCA* carriers who did not undergo CPM.^20^^,^^34^^,^^35^ Among Hodgkin lymphoma survivors, the increased risk of breast cancer is well documented, but too few patients have developed contralateral disease to make a reliable estimate of either CBC risk or CPM benefit.^36^^,^^37^

##### Summary

CPM does not appear to be associated with a survival benefit, with the possible exception of *BRCA* carriers.

### CPM’s Impact on Noncancer Outcomes

#### Surgical Risks of CPM (with/without Reconstruction)

Complications can be divided into categories based on time (early, intermediate, late) and type (surgical, oncologic, reconstructive). Early complications are dominated by surgical site issues such as tissue/skin flap necrosis, infection, bleeding, and standard surgical complications such as deep-vein thrombosis and anesthesia complications. Because reconstructive complications on the CPM side would not have occurred without the CPM, it is reasonable to consider the complications from the mastectomy and also any reconstructive complications (implant loss, flap loss, unanticipated revisions, etc.) from both the immediate procedure and subsequent procedures required for completion of the reconstruction. Taking these together, complications are estimated to occur in 40–64 % over the entire course of a reconstruction, with an estimate of 52 % of patients having at least one unanticipated surgery in one series.^38^

CPM has been shown to have double the complication rate compared to unilateral mastectomy, regardless of whether reconstruction is performed or not, and complications occur almost equally on the affected and prophylactic sides.^39^^–^42 CPM increases complications that require return to the operating room, and removal of an implant or significant skin necrosis, as well as more minor complications such as cellulitis, small areas of skin necrosis, or hematomas that do not require a return to the operating room.^39^ Potential comorbidities that may increase the likelihood of complications (such as cardiac/pulmonary comorbidities, obesity, diabetes, smoking, use of steroids or anticoagulants) should be considered when considering the possible harms of CPM and any potential benefits.

##### Summary

CPM doubles the risk of surgical complications.

#### Oncologic Risks of CPM

CPM may negatively affect oncologic outcomes for patients who were never destined to develop a CBC. Surgical complications may delay the onset of adjuvant therapy, or may encourage patients to avoid therapy that would otherwise be recommended, such as radiotherapy. One study has shown that patients undergoing CPM had longer delays to adjuvant therapy then those patients undergoing unilateral mastectomy or lumpectomy.^43^

#### CPM and Aesthetic Outcome

Symmetry is a significant driver for CPM, especially for women with ptotic or large breasts. Satisfaction with breast appearance has been shown to be higher with bilateral mastectomy and implant reconstruction compared to unilateral mastectomy with implant reconstruction.^44^ Regardless of technique, reconstructing both sides at the same time allows for more degrees of freedom and less constraints in reconstructive options, perhaps resulting in better outcomes. When autologous choices are made for reconstruction, CPM also allows for the use of the entire abdominal donor site as opposed to discarding half when a unilateral reconstruction is performed. With advances in reconstructive surgical techniques and options, CPM in patients undergoing mastectomy for their primary tumor may improve symmetry; however, it is important to educate women that symmetry procedures are available that do not require mastectomy and can provide symmetry with potentially fewer surgical and wound complications and preserve sensation.

CPM has been associated with negative outcomes on quality of life associated with reconstructive surgery. Survey studies of patients who have undergone reconstruction have shown that up to 20–30 % of patients report that outcomes from their reconstruction such as the cosmetic appearance, numbness or tingling, and sexuality were worse than expected.^45^^–^48 Additionally, a similar proportion of patients report that the number of operations needed to complete their reconstruction was more than expected, and the need for unplanned reoperations was associated with less satisfaction with the procedure.^45^^,^^49^ Patients who undergo CPM do rate their satisfaction with their breast appearance higher with reconstruction than those who do not undergo CPM, but physical effects such as pain and sexuality were no different between CPM and non-CPM patients.^44^^,^^50^ These factors, along with patient desire to improve overall aesthetic appearance of their reconstruction, should be taken into account when discussing the option of CPM and reconstruction with patients.

##### Summary

Bilateral reconstruction may provide improved cosmetic outcome.

### When CPM Should Be Considered or Discouraged

Average-risk women with unilateral breast cancer do not derive any oncologic benefit from CPM, and thus CPM should be discouraged.

From a risk perspective, CPM should be considered primarily for women at the highest risk for CBC, specifically those women with (1) deleterious mutations of *BRCA1/2,* (2) a greater than 25 % lifetime risk of breast cancer primarily due to family history in the absence of deleterious mutations, or (3) a history of mantle radiation (typically for Hodgkin lymphoma) before age 30.^36^^,^^37^

For patients with a strong family history without a known genetic mutation and patients with other breast cancer risk genes such as *CHEK2, PALB2,* and *CDH1,* there is insufficient evidence regarding CBC risk to recommend for or against CPM in these patients, and therefore CPM can be considered.

Symmetry is a significant driver for CPM, especially for those with ptotic or large breasts and unilateral breast malignancy. Plastic surgical consultation is highly recommended whenever mastectomy is required or chosen for management of the index cancer and the patient is interested in reconstruction. This consultation should involve a detailed discussion about what each reconstructive procedure involves, recovery time, the need for additional operations, and the risks associated with the procedure.

Patients with more advanced disease, when timing of adjuvant oncologic therapy is critical and risk of recurrence from the primary tumor greatly outweighs potential risk of CBC, should not undergo CPM. Additionally, patients with comorbidities such as significant cardiac or pulmonary disease, chronic anticoagulation or steroid use, poorly controlled diabetes, poor wound healing, and liver failure should be discouraged from CPM because the surgical risks of the procedure likely outweigh any benefits.

## Conclusions

CPM should be considered for those at significant risk of CBC

- Documented *BRCA1/2* carrier.
- Strong family history, but patient has not undergone genetic testing.
- History of mantle chest radiation before age 30 years.

CPM can be considered for those at lower risk of CBC

- Gene carrier of non-*BRCA* gene (e.g., *CHEK*-*2, PALB2, p53, CDH1*).
- Strong family history, patient *BRCA* negative, no known *BRCA* family member.

CPM may be considered for other reasons

- To limit contralateral breast surveillance (dense breasts, failed surveillance, recall fatigue).
- To improve reconstructed breast symmetry.
- To manage risk aversion.
- To manage extreme anxiety. (This may be better managed through psychological support strategies.)

CPM should be discouraged

- Average-risk woman with unilateral breast cancer.
- Women with advanced index cancer (e.g., inflammatory breast cancer, T4 or N3 disease, stage IV disease).
- Women at high risk for surgical complications (e.g., patients with comorbidities: obesity, smoker, diabetes).
- Woman tested *BRCA* negative with a family of *BRCA*-positive carriers.
- Male breast cancer, including *BRCA* carriers.
